# Supplementary material for: Attitude, self-efficacy, and perceived risk toward seasonal influenza vaccination among primary care physicians in Qatar: A cross-sectional study
Source: PLoS One. 2026 Jan 2;21(1):e0323518. doi: 10.1371/journal.pone.0323518 (PMC12758819; doi:10.1371/journal.pone.0323518)

PLOS ONE

Assessing attitude, self-efficacy, and perceived risk toward seasonal influenza vaccination among primary care physicians in Qatar: A cross-sectional study

--Manuscript Draft--

**Manuscript Number:**	PONE-D-25-15876
**Article Type:**	Research Article
**Full Title:**	Assessing attitude, self-efficacy, and perceived risk toward seasonal influenza vaccination among primary care physicians in Qatar: A cross-sectional study
**Short Title:**	Attitude, self-efficacy, and perceived risk of flu vaccination among primary care physicians
**Corresponding Author:**	Marwa Bibars<br>Egypt Ministry of Health and Population<br>Al haram, Giza EGYPT
**Keywords:**	Seasonal influenza, Qatar, Vaccine, Attitude, Self-efficacy, Risk, Physicians
**Abstract:**	This cross-sectional study examined influenza-related attitude, self-efficacy and perceived risk among 190 primary health care physicians. Participants were predominantly male (over 50%), aged ≥45 years (55.7%), with one-third reporting chronic diseases. Most (96.3%) had prior influenza vaccination, though only 73.3% were vaccinated in the last season. While 87.9% endorsed vaccination for healthcare workers to ensure service continuity, only 37.4% supported mandatory policies. Notably, 46% believed healthcare professionals do not pose influenza transmission risks to patients. Self-efficacy for vaccination was strongly tied to time availability (73.7% agreement) and institutional vaccine provision (78.9%), with social support from colleagues (79.5%) and relatives (68.9%) further influencing adherence. Male physicians (87.5%) and those with ≥5 prior vaccine doses (88.6%) or recent vaccination (87.3%) reported higher self-efficacy, though chronic disease history showed no significant association. Risk perception disparities emerged: 94.2% acknowledged elevated occupational risk during epidemics, yet only 62.1% perceived personal risk. Similarly, 86.3% viewed influenza as dangerous for patients versus 64.2% for themselves. Higher perceived risk scores correlated with chronic disease history (84.5), prior vaccination (81.1), recent vaccination (82.8), and ≥5 vaccine doses (85.0). Information sources prioritized official health agencies (Ministry of Public Health: 59.5%; WHO/CDC: 56.3%), while traditional media were least utilized (7.9–21.1%). These findings highlight gaps between professional risk acknowledgment and personal risk mitigation, underscoring the need for targeted strategies to address vaccine hesitancy, improve access, and align perceptions with evidence-based practices in healthcare settings.
**Order of Authors:**	Kamran Aziz
	Mansoura salem Ismail
	Marwa Bibars
	Nagah Selim
	Ayah Mohamed
	Ahmed Sameer Alnuaimi
	Muna Mehdar AlSaadi
**Additional Information:**	
**Question**	**Response**
**Financial Disclosure**<br><br>Enter a financial disclosure statement that describes the sources of funding for the	The author(s) received no specific funding for this work.

Powered by Editorial Manager® and ProduXion Manager® from Aries Systems Corporation

work included in this submission. Review the submission guidelines for detailed requirements. View published research articles from *PLOS ONE* for specific examples.<br><br>This statement is required for submission and **will appear in the published article** if the submission is accepted. Please make sure it is accurate.<br><br>**Funded studies**<br>Enter a statement with the following details:<br>• Initials of the authors who received each award<br>• Grant numbers awarded to each author<br>• The full name of each funder<br>• URL of each funder website<br>• Did the sponsors or funders play any role in the study design, data collection and analysis, decision to publish, or preparation of the manuscript?<br><br>Did you receive funding for this work?	
**Competing Interests**<br><br>Use the instructions below to enter a competing interest statement for this submission. On behalf of all authors, disclose any competing interests that could be perceived to bias this work—acknowledging all financial support and any other relevant financial or non-financial competing interests.<br><br>This statement is required for submission and **will appear in the published article** if the submission is accepted. Please make sure it is accurate and that any funding sources listed in your Funding Information later in the submission form are also declared in your Financial Disclosure statement.<br><br>View published research articles from *PLOS ONE* for specific examples.	The authors have declared that no competing interests exist.

**NO authors have competing interests**<br><br>Enter: *The authors have declared that no competing interests exist.*<br><br>**Authors with competing interests**<br><br>Enter competing interest details beginning with this statement:<br><br>*I have read the journal's policy and the authors of this manuscript have the following competing interests: [insert competing interests here]*<br><br>* typeset	
**Ethics Statement**<br><br>Enter an ethics statement for this submission. This statement is required if the study involved:<br><br>• Human participants<br>• Human specimens or tissue<br>• Vertebrate animals or cephalopods<br>• Vertebrate embryos or tissues<br>• Field research<br><br>Write "N/A" if the submission does not require an ethics statement.<br><br>General guidance is provided below. Consult the submission guidelines for detailed instructions. **Make sure that all information entered here is included in the Methods section of the manuscript.**	The Institutional Review Board at Primary Health Care Corporation in Qatar approved this study (Reference number PHCC/DCR/ 2020/06/047<br>An online survey of all primary healthcare physicians working in primary healthcare corporation in Qatar was conducted from October 11, 2021, to October 31, 2021. This was an anonymous, online-based survey utilizing implied informed consent. Participants were informed that by submitting the completed questionnaire, they were providing their consent to participate. A survey link was distributed to all physicians, accompanied by a statement indicating that clicking the link signified voluntary consent to take part in the study. The questionnaire included a cover page outlining the study's aims and objectives, emphasizing that participation was entirely voluntary and that no identifiable information would be collected. All anonymized responses were received directly by the researcher.

Powered by Editorial Manager® and ProduXion Manager® from Aries Systems Corporation

**Format for specific study types**<br><br>**Human Subject Research (involving human participants and/or tissue)**<br>• Give the name of the institutional review board or ethics committee that approved the study<br>• Include the approval number and/or a statement indicating approval of this research<br>• Indicate the form of consent obtained (written/oral) or the reason that consent was not obtained (e.g. the data were analyzed anonymously)<br><br>**Animal Research (involving vertebrate animals, embryos or tissues)**<br>• Provide the name of the Institutional Animal Care and Use Committee (IACUC) or other relevant ethics board that reviewed the study protocol, and indicate whether they approved this research or granted a formal waiver of ethical approval<br>• Include an approval number if one was obtained<br>• If the study involved *non-human primates*, add *additional details* about animal welfare and steps taken to ameliorate suffering<br>• If anesthesia, euthanasia, or any kind of animal sacrifice is part of the study, include briefly which substances and/or methods were applied<br><br>**Field Research**<br><br>Include the following details if this study involves the collection of plant, animal, or other materials from a natural setting:<br>• Field permit number<br>• Name of the institution or relevant body that granted permission	
**Data Availability**<br><br>Authors are required to make all data underlying the findings described fully available, without restriction, and from the time of publication. PLOS allows rare exceptions to address legal and ethical concerns. See the PLOS Data Policy and FAQ for detailed information.	Yes - all data are fully available without restriction

Powered by Editorial Manager® and ProduXion Manager® from Aries Systems Corporation

A Data Availability Statement describing where the data can be found is required at submission. Your answers to this question constitute the Data Availability Statement and **will be published in the article**, if accepted.<br><br>**Important:** Stating 'data available on request from the author' is not sufficient. If your data are only available upon request, select 'No' for the first question and explain your exceptional situation in the text box.<br><br>Do the authors confirm that all data underlying the findings described in their manuscript are fully available without restriction?	
**Describe where the data may be found in full sentences. If you are copying our sample text, replace any instances of XXX with the appropriate details.**<br><br>• If the data are **held or will be held in a public repository**, include URLs, accession numbers or DOIs. If this information will only be available after acceptance, indicate this by ticking the box below. For example: *All XXX files are available from the XXX database (accession number(s) XXX, XXX.)*.<br>• If the data are all contained **within the manuscript and/or Supporting Information files**, enter the following: *All relevant data are within the manuscript and its Supporting Information files.*<br>• If neither of these applies but you are able to provide **details of access elsewhere**, with or without limitations, please do so. For example:<br><br>*Data cannot be shared publicly because of [XXX]. Data are available from the XXX Institutional Data Access / Ethics Committee (contact via XXX) for researchers who meet the criteria for access to confidential data.*<br><br>*The data underlying the results presented in the study are available from (include the name of the third party*	All relevant data are within the manuscript and its Supporting Information files.

and contact information or URL).<br>• This text is appropriate if the data are owned by a third party and authors do not have permission to share the data.<br><br>* typeset	
Additional data availability information:	

Manuscript
Click here to access/download;Manuscript;Manuscript.docx

Assessing attitude, self-efficacy, and perceived risk toward seasonal influenza vaccination among primary care physicians in Qatar: A cross-sectional study

Authors

Kamran Aziz [1&5] Mansoura Salem Ismail [1,2&5] Marwa Bibars [3*] Nagah Selim[1&4] Ayah Mohamed[6], Ahmed Sameer Alnuaimi[1] Muna Mehdar AlSaadi [1]

Affiliations:
1-Primary Health Care Corporation, Doha, Qatar
2-Family Medicine, Suez Canal University Faculty of Medicine, Ismailia, Egypt
3- Ministry of Health and Population, Giza, Egypt
4- Public health and preventive medicine, Cairo University Faculty of Medicine, Egypt
5-Qatar University, College of Medicine, Qatar
6- University of the Pacific, California, USA

* Corresponding author

Email: bibars.marwa@gmail.com

1. Abstract

This cross-sectional study examined influenza-related attitude, self-efficacy and perceived risk among 190 primary health care physicians. Participants were predominantly male (over 50%), aged ≥45 years (55.7%), with one-third reporting chronic diseases. Most (96.3%) had prior influenza vaccination, though only 73.3% were vaccinated in the last season. While 87.9% endorsed vaccination for healthcare workers to ensure service continuity, only 37.4% supported mandatory policies. Notably, 46% believed healthcare professionals do not pose influenza transmission risks to patients. Self-efficacy for vaccination was strongly tied to time availability (73.7% agreement) and institutional vaccine provision (78.9%), with social support from colleagues (79.5%) and relatives (68.9%) further influencing adherence. Male physicians (87.5%) and those with ≥5 prior vaccine doses (88.6%) or recent vaccination (87.3%) reported higher self-efficacy, though chronic disease history showed no significant association. Risk perception disparities emerged: 94.2% acknowledged elevated occupational risk during epidemics, yet only 62.1% perceived personal risk. Similarly, 86.3% viewed influenza as dangerous for patients versus 64.2% for themselves. Higher perceived risk scores correlated with chronic disease history (84.5), prior vaccination (81.1), recent vaccination (82.8), and ≥5 vaccine doses (85.0). Information sources prioritized official health agencies (Ministry of Public Health: 59.5%; WHO/CDC: 56.3%), while traditional media were least utilized (7.9–21.1%). These findings highlight gaps between professional risk acknowledgment and personal risk mitigation, underscoring the need for targeted strategies to address vaccine hesitancy, improve access, and align perceptions with evidence-based practices in healthcare settings.

2. Introduction

Seasonal influenza is considered a significant public health concern that leads to sizable morbidity, mortality, and healthcare costs worldwide. It is projected that there are about one billion cases of influenza worldwide per year, of which 3–5 million are severe cases and nearby 0.65 million lead to influenza-related respiratory death [1,2]. Health workers (HWs) are at increased risk of influenza virus infection, which may vary depending on occupation or setting [3]. Infected HWs could increase the risk of nosocomial infection and community spread [4,5]. The seasonal influenza vaccine is one of the most effective preventive measures to reduce the impact of influenza infections. The World Health Organization (WHO) considers HWs to be a priority group for influenza vaccination and reinforces this position by supporting countries to develop and implement national, seasonal immunization policies for HWs, but vaccination rates among healthcare workers, remain suboptimal. Primary care physicians (PCPs) play a critical role in influencing vaccination decisions, both for their patients and for themselves. However, the success of vaccination programs often depends on the attitudes, perceived risks, and self-efficacy of healthcare providers, including PCPs. [6,9]. PCPs' attitudes toward the seasonal influenza vaccine can influence their recommendation and administration of the vaccine to their patients. Studies suggest that healthcare professionals' personal attitudes and beliefs about vaccines are significant predictors of their vaccination practices. Positive attitudes toward vaccination can increase vaccination rates. PCPs' perception of the risks associated with influenza—also plays a crucial role in vaccination uptake [10,12]. Research has shown that healthcare workers who perceive themselves at higher risk for contracting or transmitting influenza are more likely to get vaccinated. Self-efficacy, or the belief in one's ability to execute a behavior successfully, is another key factor influencing vaccination practices. PCPs with high self-efficacy regarding the influenza vaccine are more likely to recommend it to patients and receive it themselves. Self-efficacy can be shaped by various factors, including training, knowledge of the vaccine's benefits, and previous positive experiences with

vaccination. Enhancing PCPs' self-efficacy through education and training can lead to higher vaccine uptake rates [13,15]. Understanding PCPs' attitudes, perceived risks, and self-efficacy regarding the seasonal influenza vaccine is vital for improving vaccination coverage. Effective vaccination campaigns must address the factors that influence healthcare providers' vaccine behaviors, as they serve as trusted sources of information for patients. By focusing on improving attitudes and self-efficacy, public health initiatives can enhance influenza vaccination rates, ultimately reducing the burden of influenza-related morbidity and mortality.

3. Method of study

An online survey of all primary healthcare physicians working in primary healthcare corporation in Qatar was conducted from October 11, 2021, to October 31, 2021.

A validated questionnaire was obtained from Asma et al. [16] study about background characteristics such as age, gender, working experience in primary care, and history of influenza vaccination… The second section included attitude, self-efficacy, and perceived risk regarding influenza vaccination, which were assessed using five-point Likert questions. Responses were expressed as follows: 1 means totally agree, 2 means I agree, 3 means neutral, 4 means I disagree, and 5 means I totally disagree. The percentage reported was for those who answered as agree or strongly agree and each positive item was awarded a score of one point. The total score was calculated by summing the scores of positive items and multiplying it by 100/maximum count of items.

3.1 Data analysis

The collected data was analyzed using SPSS version 25 and the quality of the data was checked before proceeding to the analysis. Quantitative data was summarized in the form of mean and SD while qualitative data was summarized in the form of frequency tables and charts. The

Bivariate analysis was then carried out using the independent t-test for two groups and ANOVA for more than two groups to investigate the association between the dependent variables and the background characteristics of physicians. Effect size is assessed by Cohen's d for differences in the mean between two groups, while Eta squared test was used for the effect of a factor with more than two groups. The level of statistical significance was set at a p-value of less than or equal to 0.05.

3.2 Ethics statements

This was an anonymous, online-based survey utilizing implied informed consent. Participants were informed that by submitting the completed questionnaire, they were providing their consent to participate. A survey link was distributed to all physicians, accompanied by a statement indicating that clicking the link signified voluntary consent to take part in the study. The questionnaire included a cover page outlining the study's aims and objectives, emphasizing that participation was entirely voluntary and that no identifiable information would be collected. All anonymized responses were received directly by the researcher.

3.3 Ethics approval

The Institutional Review Board at Primary Health Care Corporation in Qatar approved this study (Reference number PHCC/DCR/ 2020/06/047

4. Results

The overall response rate in this study was 42%, 106 (55.7%) were 45 years old and above, and more than half of the participants were males. About one-third reported a history of Chronic diseases [Table 1].

Table 1: background characteristics of physicians working in Primary healthcare centers in Qatar.

Characteristic	N	%
Age		
≤44	84	44.2
≥ 45	106	55.7
Female	78	41.1
Male	112	58.9
Duration of PHCC work experience in years		
<8	134	70.5
≥8	56	29.5
History of chronic diseases		
No	129	67.9
Yes	61	32.1
Total	190	100

Most participants (96.3%) had been vaccinated against influenza in the past, while 73.3% of the physicians had received an influenza vaccine in the last season [Table 2].

Table 2: History of Influenza vaccine uptake among primary care healthcare in Qatar.

Ever taken influenza vaccination		
No	7	3.7
Yes	183	96.3
influenza vaccination last season		
No	50	26.3
Yes	140	73.7
Doses of seasonal influenza vaccine		
Never	7	3.7
(1-2)	25	13.2
(3-4)	49	25.8
≥5	87	45.8
Don't remember	22	11.6
Total	190	100.0

Of 190 doctors, the majority (87.9%) believe that health professionals should be vaccinated for the continuity of health services. Despite this, only 37.4% believe that the influenza vaccine should be mandatory for healthcare workers. Notably, nearly 46% of the respondents feel confident that health professionals do not pose a risk of spreading the influenza virus to their patients [Table 3].

Table 3: Attitude of primary care physicians in Qatar towards Influenza vaccination (*N* =190).

	n	%	95%confidence interval
I feel that health professionals are not spreading the disease to their patients	87	45.8	(38.8-52.9)
I believe that health professionals should be vaccinated for the continuity of health services	167	87.9	(82.7-92)
Influenza vaccine should be mandatory for health professionals	71	37.4	(30.7-44.4)

When asked about their self-efficacy in successfully getting vaccinated, Time and availability were the two most important factors. 73.7% agreed that they would be vaccinated if they had enough time while 78.9% expressed willingness to be vaccinated if the institute provided the vaccine. Perceived social support for influenza vaccination from relatives and colleagues was frequently reported with individual self-efficacy (68.9% & 79.5% respectively) [Table 4].

Table 4: Self-efficacy of primary care physicians towards Influenza vaccination (*N* =190).

	n	%	95%confidence interval
My relatives believe that my vaccination is important	131	68.9	(62.1 - 75.2)
My institute recommends being vaccinated	185	97.4	(94.3 - 99)
My colleagues believe that my vaccination is important	151	79.5	(73.3 - 84.7)
The Ministry of Health recommends vaccination of health professionals	187	98.4	(95.8 - 99.6)
The health authorities I know recommend vaccination	182	95.8	(92.2 - 98)

I would be vaccinated every year if I have enough time	140	73.7	(67.1 - 79.6)
I would be vaccinated if someone reminds me	126	66.3	(59.4 - 72.7)
I would be vaccinated every year if the vaccine is provided in my institute	150	78.9	(72.7 - 84.3)
I would be vaccinated every year if I am rewarded	64	33.7	(27.3 - 40.6)
I would be vaccinated for influenza every year if sufficient knowledge was given	125	65.8	(58.8 - 72.3)

Significant positive associations with self-efficacy mean scores were observed among primary care physicians who were male (87.5%) and those who had received five or more influenza vaccinations (88.6%), particularly in the most recent season (87.3%). In contrast, there was no statistically significant relationship between a history of chronic diseases and self-efficacy scores [Table 5].

Table 5: Relationship between sociodemographics of primary care physicians and perceived self-efficacy score in Qatar 2021 (*N* =190).

	Perceived Self efficacy Score						
**Characteristic**	**Range**	**Mean**	**SD**	**SE**	**Physicians' frequency**	***p*-Value**	**Effect size**
Age (years)							
22-34	(78-96)	86.0	5.4	1.64	11	0.91	0.003
35-44	(58-110)	85.0	11.9	1.39	73		
45-54	(68-106)	86.3	10.0	1.16	74		
≥55	(56-110)	85.3	11.6	2.05	32		
Gender							
Female	(58-110)	83.0	11.0	1.25	78	0.005	-0.424
Male	(56-110)	87.5	10.3	0.97	112		
Duration of PHCC work experience in years							
<4	(68-110)	85.8	10.0	1.31	58	0.55	0.011

4-7	(56-108)	85.4	11.6	1.33	76		
8-10	(68-110)	88.8	10.6	2.51	18		
≥11	(62-108)	84.3	10.5	1.70	38		
History of chronic disease							
Negative	(58-110)	85.4	11.1	0.98	129	0.7	-0.058
Positive	(56-104)	86.1	10.2	1.31	61		
Ever taken the influenza vaccine							
Negative	(58-84)	76.3	9.7	3.66	7	0.038	-0.912
Positive	(56-110)	86.0	10.7	0.79	183		
Influenza vaccination last season							
Negative	(58-110)	81.0	11.3	1.60	50	<0.001	-0.598
Positive	(56-110)	87.3	10.1	0.86	140		
Doses of seasonal influenza vaccine received							
Never	(58-84)	76.3	9.7	3.66	7	<0.001	0.098
(1-2)	(58-102)	80.4	11.0	2.19	25		
(3-4)	(68-106)	85.0	9.0	1.29	49		
≥5	(56-110)	88.6	11.1	1.19	87		

In assessing risk perception, a difference was reported between physicians' acknowledgment of risk and their personal risk perception. While 94.2% agreed healthcare professionals face the greatest influenza risk during epidemics, only 62.1% believed they were at high risk. This

was reflected in perceived severity; 86.3% considered influenza dangerous for patients, versus only 64.2% for themselves [Table 6].

Table 6: Seasonal influenza vaccine perceived risk and severity of risk among primary care physicians (*N* =190).

	n	%	95%confidence interval
Perceived risk			
I'm at high risk for influenza	118	62.1	(55.1 - 68.8)
I can spread infection to my patients even if I am asymptomatic	153	80.5	(74.5 - 85.7)
Health professionals are under the highest risk in case of an epidemic	179	94.2	(90.2 - 96.9)
I can spread infection to my family even if I am asymptomatic	170	89.5	(84.5 - 93.2)
Perceived severity of risk			
Influenza is dangerous for me	122	64.2	(57.2 - 70.8)
Influenza is dangerous for my patients	164	86.3	(80.9 - 90.6)
Influenza is dangerous for my family	148	77.9	(71.6 - 83.3)

The analysis revealed a statistically significant association between physicians' perceived risk mean scores and select background characteristics. Notably, higher mean scores were observed among those with a history of chronic diseases (84.5), prior influenza vaccination (81.1), influenza vaccination in the last season (82.8), and having received five or more seasonal influenza vaccine doses (85.0) [Table 7].

Table 7: Relationship between sociodemographic of primary care physicians and perceived risk score in Qatar 2021 (*N* =190).

**Characteristic**	**Range**	**Mean**	**SD**	**SE**	**Physicians' frequency**	***p*-Value**	**Effect size**
Age (years)						0.18[NS]	0.026
22-34	(40 - 100)	75.6	15.3	4.61	11		
35-44	(49 - 100)	79.1	12.8	1.5	73		
45-54	(46 - 100)	82.5	12.4	1.44	74		
≥55	(60 - 100)	82.1	11.5	2.03	32		
Gender						0.24[NS]	-0.176
Female	(40 - 100)	79.4	13.2	1.49	78		

Male	(46 - 100)	81.6	12.2	1.16	112		
Duration of PHCC work experience in years						0.45[NS]	0.014
<4	(40 - 100)	78.9	13.1	1.73	58		
4-7	(46 - 100)	80.8	12.7	1.46	76		
8-10	(57 - 100)	84	12.5	2.94	18		
≥11	(60 - 100)	81.8	11.7	1.9	38		
Having chronic disease						0.70[NS]	-0.058
Negative	(40 - 100)	78.9	13.2	1.16	129		
Positive	(63 - 100)	84.5	10.5	1.35	61		
Positive history of influenza vaccination						<0.001	-0.912
Negative	(63 - 77)	71	6.5	2.45	7		
Positive	(40 - 100)	81.1	12.7	0.94	183		
Positive history of influenza vaccination last season						<0.001	-0.640
Negative	(40 - 100)	75	13.5	1.91	50		
Positive	(46 - 100)	82.8	11.7	0.99	140		
Count of seasonal influenza vaccine doses received						<0.001	0.134
Never	(63 - 77)	71	6.5	2.45	7		
(1-2)	(40 - 94)	72.5	14.4	2.88	25		
(3-4)	(51 - 100)	79.2	12.7	1.81	49		
≥5	(51 - 100)	85	10.7	1.15	87		

Physicians primarily rely on official websites from the Ministry of Public Health (59.50%), WHO, and CDC (56.30%) for influenza vaccine updates. Conversely, newspapers (21.10%), TV, and flu-specific platforms (7.90%) are the least consulted sources [Fig. 1].

Fig.1: Bar chart for the source of information.

Discussion

The study focused on primary care physicians' attitudes, self-efficacy, and perceived risk regarding accepting the seasonal influenza vaccine.

The study revealed that most participants had received an influenza vaccination in the past, while two-thirds of physicians had been vaccinated during the last season. This is per a study done in the US, where family physicians’ vaccination rate was up to 87%, attributed to workplace policies and free access on-site for all staff. [17].

Most physicians believe that healthcare professionals should be vaccinated to ensure the continuity of health services. Studies have shown that physicians know their occupational obligations towards vaccination [18,19].

According to Polan et al. when healthcare professionals are vaccinated against sessional influenza, they increase the ability of the healthcare system to respond effectively to influenza epidemics [20].

About one-third of the physicians thought the influenza vaccine should be mandatory for healthcare professionals. According to Winston et al. mandatory vaccination increases vaccination rates but healthcare professionals negatively perceive it [21].

Approximately half of the respondents felt that health professionals do not contribute to the spread of the influenza virus to their patients. However, some studies suggest that the disease is often asymptomatic, and healthcare professionals can spread the influenza virus to their patients and families [22-24].

The availability of the influenza vaccine and the time needed to get vaccinated were crucial factors influencing individuals' self-efficacy regarding vaccine uptake. Approximately two-thirds of participants indicated that they would be willing to get vaccinated if they had enough time and access to the vaccine at their institution. AlMarzooqi et al. reported that one of the frequently cited reasons for not taking influenza vaccine was lack of time to take the vaccine (28.9%). The majority (85.1%) of healthcare professionals received the vaccine from the governmental sector, which was also their workplace [25].

According to a study by Cowan et al. in the US, the lack of time (34%) was a common reason for not receiving the vaccine [17].

According to Wallace et al. 5–60% of the healthcare workers reported time constraints for rejection of the influenza vaccine [26].

Additionally, social support from family and colleagues plays a significant role in enhancing a person's self-efficacy. Takayanagi et al. showed that “believing that most departmental colleagues had been vaccinated” ($P < .0001$) was significantly associated with compliance with influenza vaccination [24].

Among male primary care physicians, those who had received five or more influenza vaccinations in the past showed a significant positive association with self-efficacy.

Alenazi et al. found that increasing age, longer work duration in health services, being male and being a physician, are associated with significantly larger vaccination compliance ($p=0.02$, $p=0.07$, $p=0.01$, $p=0.01$) respectively [27].

The survey found no statistically significant relationship between having a history of chronic diseases and self-efficacy. This is contrary to a study by AlMarzooqi et al. where it was noted that participants with diabetes, bronchial asthma and obesity had increased acceptance of the influenza vaccine ($P < .001$) [25].

There is a notable difference in how physicians acknowledge risk versus their perception of risk. Most physicians agree that healthcare professionals are at the highest risk for influenza during epidemics, however, fewer believe they are at high risk. Studies have found that one main reason for not receiving seasonal influenza vaccine among healthcare professionals was that they did not recognize themselves as at risk [28,29].

According to AlMarzooqi et al. the most common reason among health care professionals for not being vaccinated was their belief of not at high risk of contracting influenza [25].

The study revealed that physicians primarily depend on official sources for information about the influenza vaccine, such as the Ministry of Public Health, WHO, and CDC. In contrast, they consult electronic and print media and flu-specific resources much less frequently for influenza information. Alame et al. reported the participants relied on various sources for vaccine recommendations, including international organizations (e.g., WHO), and the Ministry of health [30].

Most healthcare professionals (70.5%) were aware of CDC recommendations concerning influenza vaccines for healthcare professionals [25].

Conclusion

This study highlights gaps between professional risk acknowledgment and personal risk mitigation, underscoring the need for targeted strategies to address vaccine hesitancy, improve access, and align perceptions with evidence-based practices in healthcare settings

Acknowledgments

The authors of this manuscript would like to thank primary care physicians in Qatar who participated in this survey.

References

1.Iuliano A.D., Roguski K.M., Chang H.H., Muscatello D.J., Palekar R., Tempia S., Cohen C., Gran J.M., Schanzer D., Cowling B.J., et al. Estimates of global seasonal influenza-associated respiratory mortality: A modelling study. Lancet. 2018;391:1285–1300. doi: 10.1016/S0140-6736(17)33293-2.]

2.Paget J., Spreeuwenberg P., Charu V., Taylor R.J., Iuliano A.D., Bresee J., Simonsen L., Viboud C. Global mortality associated with seasonal influenza epidemics: New burden estimates and predictors from the GLaMOR Project. J. Glob. Health. 2019;9:020421. doi: 10.7189/jogh.09.020421.

3.Lietz J., Westermann C., Nienhaus A., Schablon A. The Occupational Risk of Influenza A (H1N1) Infection among Healthcare Personnel during the 2009 Pandemic: A Systematic Review and Meta-Analysis of Observational Studies. PLoS ONE. 2016;11:e0162061. doi: 10.1371/journal.pone.0162061

4.Srinivasan A., Perl T.M. Respiratory protection against influenza. JAMA. 2009;302:1903–1904. doi: 10.1001/jama.2009.1494.

5.Kuster S.P., Shah P.S., Coleman B.L., Lam P.P., Tong A., Wormsbecker A., McGeer A. Incidence of influenza in healthy adults and healthcare workers: A systematic review and meta-analysis. PLoS ONE. 2011;6:e26239. doi: 10.1371/journal.pone.0026239.

6.Durando P., Alicino C.A.-O., Dini G., Barberis I., Bagnasco A.M., Iudici R., Zanini M., Martini M., Toletone A., Paganino C., et al. Determinants of adherence to seasonal influenza vaccination among healthcare workers from an Italian region: Results from a cross-sectional study. BMJ Open. 2016;6:e010779. doi: 10.1136/bmjopen-2015-010779.

7.World Health Organization Global Influenza Strategy 2019–2030. [(accessed on 5 March 2020)]. Available online: https://apps.who.int/iris/handle/10665/311184.

8.World Health Organization Vaccines against influenza WHO position paper—November 2012. Wkly. Epidemiol. Rec. 2012;87:461–476.

9.Dini G., Toletone A., Sticchi L., Orsi A., Bragazzi N.L., Durando P. Influenza vaccination in healthcare workers: A comprehensive critical appraisal of the literature. Hum. Vaccin. Immunother. 2018;14:772–789. doi: 10.1080/21645515.2017.1348442.

10. Green, M. S., & Spivak, L. (2015).* The role of healthcare providers in influencing vaccine acceptance. *Vaccine, 33*(46), 6013-6019. DOI : 10.1016/j.vaccine.2015.09.019

11. McGaw, K. S., & Thompson, L. (2017).* Healthcare providers' attitudes and vaccination behavior: A systematic review of the literature. *Vaccine, 35*(33), 4293-4300. DOI : 10.1016/j.vaccine.2017.06.063

12. Tartof, S. Y., & McWilliams, D. (2019).* The impact of training on primary care physicians' vaccination practices: A review. *The Journal of Primary Care & Community Health, 10*, 2150132719863690. DOI : 10.1177/2150132719863690

13. Al-Qahtani, S. H., & Al-Dubai, S. A. R. (2018).* Primary care physicians' attitudes towards influenza vaccination in the Middle East: A systematic review. *Vaccine, 36*(7), 876-883. DOI: 10.1016/j.vaccine.2017.11.090

14. Omer, S. B., & Salmon, D. A. (2017).* Vaccination attitudes among healthcare providers and their influence on influenza vaccination. *Journal of Public Health Policy, 38*(4), 484-496. DOI: 10.1057/s41271-017-0106-0

15. World Health Organization (WHO). (2020).* Influenza vaccination: An essential preventive measure. *WHO Vaccine Report*. Retrieved from https://www.who.int/influenza/vaccines/en/ These references cover studies on healthcare workers' attitudes, perceived risks, and behaviors regarding vaccination and provide background information on factors influencing vaccine uptake, including the role of education and training.

16. Asma S, Akan H, Uysal Y, Poçan AG, Sucaklı MH, Yengil E, Gereklioğlu Ç, Korur A, Başhan İ, Erdogan AF, Özşahin AK. Factors affecting influenza vaccination uptake among health care workers: a multi-center cross-sectional study. BMC Infect Dis. 2016 Dec;16(1):1-9. doi: 10.1186/s12879-016-2108-1.

17. Cowan A, Winston C, Davis M, Wortley P, Clark S. Influenza vaccination status and influenza-related perspectives and practices among US physicians. Am J Infect Control. 2006;34(4):164-169. doi: 10.1016/j.ajic.2005.09.007.

18. Paya N, Pozzetto B, Berthelot P, Vallee J, Vallée J. [Vaccination status of family physicians in the Loire district, France]. Med Mal Infect. 2013;43(6):239-43. doi: 10.1016/j.medmal.2013.05.006

19. Ward K, Seale H, Zwar N, Leask J, MacIntyre CR. Annual influenza vaccination: Coverage and attitudes of primary care staff in Australia. Influenza Other Respi Viruses. 2011;5(2):135-41. doi: 10.1111/j.1750-2659.2010.00158.x

20. Poland GA. The 2009-2010 influenza pandemic: effects on pandemic and seasonal vaccine uptake and lessons learned for seasonal vaccination campaigns. Vaccine. 2010;28 Suppl 4:D3-13. doi: 10.1016/j.vaccine.2010.08.024

21. Winston L, Wagner S, Chan S. Healthcare workers under a mandated H1N1 vaccination policy with employment termination penalty: a survey to assess employee perception. Vaccine. 2014;32(37):4786-90. doi: 10.1016/j.vaccine.2014.06.001.

22. Wilde JA, McMillan JA, Serwint J, Butta J, O'Riordan MA, Steinhoff MC. Effectiveness of Influenza Vaccine in Health Care Professionals: A Randomized Trial. JAMA. 1999;281(10):908-913. doi: 10.1001/jama.281.10.908

23. Dash GP, Fauerbach L, Pfeiffer J, Pfeiffer J, Soule B, Bartley J, et al. APIC position paper: improving health care worker influenza immunization rates. Am J Infect Control. 2004;32(3):123-5. doi: 10.1016/j.ajic.2004.01.008

24. Takayanagi IJ, Cardoso MR, Costa SF, Araya ME, Machado CM. Attitudes of health care workers to influenza vaccination: why are they not vaccinated? Am J Infect Control. 2007;35(1):56-61. doi: 10.1016/j.ajic.2006.06.002

25. AlMarzooqi LM, AlMajidi AA, AlHammadi AA, AlAli N. Knowledge, attitude, and practice of influenza vaccine immunization among primary healthcare providers in Dubai health authority, 2016-2017. Hum Vaccin Immunother. 2018;14(12):2999-3004. doi:10.1080/21645515.2018.1507667

26. Wallace L. Acceptance and Uptake of Influenza Vaccination by Health Care Workers [dissertation]. Minneapolis (MN): Walden University; 2015.

27. Alenazi BR, Hammad SM, Mohamed AE. Prevalence of seasonal influenza vaccination among primary healthcare workers in Arar city, Saudi Arabia. Electron Physician. 2018;10(8):7217-7223. doi: 10.19082/7217

28. Bonaccorsi G, Lorini C, Santomauro F, Guarducci S, Pellegrino E, Puggelli F, Balli M, Bonanni P. Predictive factors associated with the acceptance of pandemic and seasonal influenza vaccination in health care workers and students in Tuscany, Central Italy. Hum Vaccin Immunother. 2013;9(12):2603-2612. DOI: 10.4161/hv.26036

29. Amodio E, Tramuto F, Maringhini G, Asciutto R, Firenze A, Vitale F, Costantino C, Calamusa G. Are medical residents a "core group" for future improvement of influenza vaccination coverage in health-care workers? A study among medical residents at the University Hospital of Palermo (Sicily). Vaccine. 2011;29(46):8113-7. doi:10.1016/j.vaccine.2011.08.033; PMID: 21856362

30. Alame M, Kaddoura M, Kharroubi S, Ezzeddine F, Hassan G, Diab El-Harakeh M, Al Ariqi L, Abubaker A, Zaraket H. Uptake rates, knowledge, attitudes, and practices toward seasonal influenza vaccination among healthcare workers in Lebanon. Hum Vaccin Immunother. 2021;17(11):4623-4631.DOI: 10.1080/21645515.2021.1964353

 Click here to access/download;Figure;Fig 1 .tiff

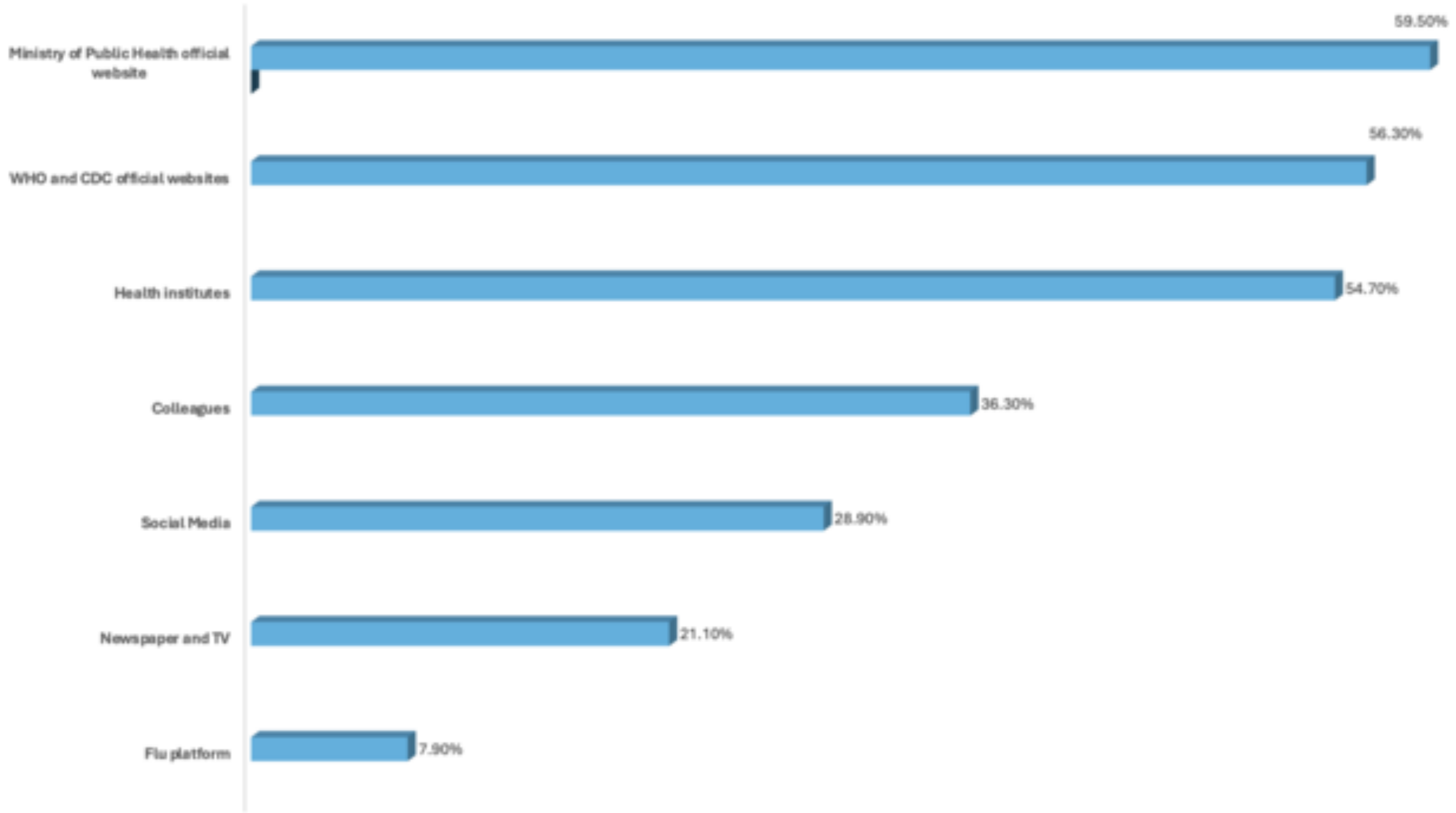

Supplement: Attachment — Submitted filename: Assessing attitudes,....pdf [file pone.0323518.s002.pdf]
