# Supplementary material for: Attitude, self-efficacy, and perceived risk toward seasonal influenza vaccination among primary care physicians in Qatar: A cross-sectional study
Source: PLoS One. 2026 Jan 2;21(1):e0323518. doi: 10.1371/journal.pone.0323518 (PMC12758819; doi:10.1371/journal.pone.0323518)
Supplement: Attachment — Submitted filename: PONE-D-25-15876 Comments.docx [file pone.0323518.s001.docx]

Title:

PONE-D-25-15876:

Assessing attitude, self-efficacy, and perceived risk toward seasonal influenza vaccination among primary care physicians in Qatar: A cross-sectional study

General comments:

Thank you for giving me the opportunity to review this manuscript. Previous studies have primarily focused on primary care doctors' attitudes toward influenza vaccine, with limited research on their self-efficacy. Therefore, this study may contribute further to the literature. However, further details are required to add clarity to the manuscript.

Specific comments as below:

Title:

I suggest removing the word ‘Assessing’ in the title to be more concise.

Introduction:

Line 61: ‘...nearby 0.65 million…’ is not clear. I suggest changing the word ‘nearby’ or rephrase.

Line 69: to add reference(s) supporting the statement ‘vaccination rates among health care workers remain suboptimal.’

The aim of the study is not clearly stated.

Method:

A brief background of primary healthcare corporation in Qatar will be useful for the reader as it is not clear whether this constitutes a chain of clinics.

More details about the questionnaire used in this study are required. Was the questionnaire adopted unchanged from Asma et al or were any modifications made? This is in view that Asma et al’s study was regarding factors affecting influenza vaccine uptake and not directly on attitude. If any translation of language was done, this need to be stated too.

If the questionnaire was adapted, whether pilot testing and validation were conducted.

How many items are there for attitude, self-efficacy and perceived risks respectively?

What is meant by ‘positive items?’

The statement: ‘each positive item was awarded a score of one point’ needs more explanation as it is not clear. How was ‘negative item’ analysed?

Line 101: it is stated that total score was calculated. However, Table 5 and 7 shows mean self-efficacy and perceived risk score. This needs to be clarified.

How were the primary healthcare physicians identified, e.g. was it through a database? How were they invited to the study, e.g. was it via emails?

What was the total number of physicians invited to participate in the study?

Were there any strategies done to maximise the response rate e.g. reminders?

Results:

Tables 1 & 2: to state the total number of respondents (N) after the title (as stated in Table 3). The last row in the table ‘Total’ is not relevant and can be removed.

Table 1: it was not stated what PHCC means.

Table 2: columns 2 & 3, there are no title to the columns, whether it describes number and percentages.

Table 3 and 4: does column ‘n’ represents the number of respondents who answered ‘agree’ and strongly agree’ to each statement (referring to the method of study)? If so, it also needs to be stated in the table.

Line 148: ‘Time’ should be written in lower case.

The items in Table 4 appears to be factors influencing influenza vaccination or factors influencing self-efficacy. Self-efficacy is assessed by determining ‘levels of confidence in performing tasks and whether or not someone can do or accomplish a given task’. This need to relate with the objectives of this study which need to be clearly stated.

Table 5 and Table 7:

The term ‘physicians’ frequency’ is confusing.

Stating the responses as ‘yes’ or ‘no’ for ‘history of chronic disease’, ‘ever taken influenza vaccine’ and ‘influenza vaccination last season’ will give more clarity compared to ‘negative’ and ‘positive’.

Written as ‘Ever taken influenza vaccine’ in Table 5, whereas in Table 7, it is written as ‘Positive history of influenza vaccination’. It is best to standardise the statements if it refers to the same questionnaire statement. Same with ‘influenza vaccination last season’.

Table 7: suggest removing ‘[NS]’.

Discussion:

Discussion can be further elaborated:

Is there a policy on influenza vaccination in the healthcare corporation of Qatar which may explain the why two thirds of the respondents had been vaccinated?

What are the possible reasons that may explain the respondents having not enough time and no access to the vaccine?

To include discussion on limitations of this study and future recommendations.

Lines 212 – 215, 220 – 224, 242 – 243: the flow of writing need to be improved as it appears disjointed.

References:

To check reference (15): the page in the link could not be found. It is not necessary to explain on what the reference is about in the reference list.
