# Supplementary material for: Attitude, self-efficacy, and perceived risk toward seasonal influenza vaccination among primary care physicians in Qatar: A cross-sectional study
Source: PLoS One. 2026 Jan 2;21(1):e0323518. doi: 10.1371/journal.pone.0323518 (PMC12758819; doi:10.1371/journal.pone.0323518)
Supplement: Attachment — Submitted filename: Response_to_Reviewers_auresp_2.docx [file pone.0323518.s006.docx]

Reviewers' comments:

Reviewer's Responses to Questions

**Comments to the Author**

1. If the authors have adequately addressed your comments raised in a previous round of review and you feel that this manuscript is now acceptable for publication, you may indicate that here to bypass the “Comments to the Author” section, enter your conflict of interest statement in the “Confidential to Editor” section, and submit your "Accept" recommendation.

Reviewer #1: (No Response)

Reviewer #2: All comments have been addressed

2. Is the manuscript technically sound, and do the data support the conclusions?

The manuscript must describe a technically sound piece of scientific research with data that supports the conclusions. Experiments must have been conducted rigorously, with appropriate controls, replication, and sample sizes. The conclusions must be drawn appropriately based on the data presented.

Reviewer #1: Yes

Reviewer #2: Yes

3. Has the statistical analysis been performed appropriately and rigorously?

Reviewer #1: Yes

Reviewer #2: Yes

4. Have the authors made all data underlying the findings in their manuscript fully available?

The [PLOS Data policy](http://track.editorialmanager.com/CL0/http:%2F%2Fwww.plosone.org%2Fstatic%2Fpolicies.action%23sharing/1/010f0199ff2bf550-1a75023e-f086-4322-8d7f-d2a752d9b63d-000000/qJimjRviBRwjVDTwOvyC1ie_er47nf-La8wSCiQF0Ps=233) requires authors to make all data underlying the findings described in their manuscript fully available without restriction, with rare exception (please refer to the Data Availability Statement in the manuscript PDF file). The data should be provided as part of the manuscript or its supporting information, or deposited to a public repository. For example, in addition to summary statistics, the data points behind means, medians and variance measures should be available. If there are restrictions on publicly sharing data—e.g. participant privacy or use of data from a third party—those must be specified.

Reviewer #1: No

Reviewer #2: Yes

An official email was sent to primary health care clinical research department and this was the response: Data underlying the findings are subject to PHCC Qatar’s data sharing policy and IRB restrictions. Aggregate data supporting the results are available within the manuscript. Individual-level data cannot be publicly shared.

5. Is the manuscript presented in an intelligible fashion and written in standard English?

PLOS ONE does not copyedit accepted manuscripts, so the language in submitted articles must be clear, correct, and unambiguous. Any typographical or grammatical errors should be corrected at revision, so please note any specific errors here.

Reviewer #1: Yes

Reviewer #2: Yes

6. Review Comments to the Author

Please use the space provided to explain your answers to the questions above. You may also include additional comments for the author, including concerns about dual publication, research ethics, or publication ethics. (Please upload your review as an attachment if it exceeds 20,000 characters)

Reviewer #1: General comments:
Thank you for giving me the opportunity to review the revised manuscript. The author had addressed all the comments. However, there are some comments for further improvement.

Specific comments as below:
Introduction:
Line 74-78: This study did not investigate strategies to improve PCPs’ attitude and self-efficacy; therefore, this sentence does not align with the study’s aims. Suggest remove or re-phrase the sentence.

-removed

Method:
Line 84-85: The word ‘physician’ is in lower case; ‘A list of physicians’ emails….’

-Done

Line 85: Write ‘PHCC’ in full at first mention and use the abbreviation from line 88 onwards.

-Done

Line 97-100: This sentence is lengthy and difficult to understand. Suggest rephrasing.

-A validated questionnaire from Asma et al. [16] was adapted with minor demographic changes, including PHCC work experience. The second section assessed attitude using 13 items, of which 10 measured self-efficacy, five on social influence and five on personal competence.

Line 102: To improve clarity, suggest amending: ‘….five-point Likert scale questions’.

-Done

Line 119: Is there a missing sentence after ‘The…’?

-Removed

Line 128: The letter ‘a’ is lower case in this sentence; ….a survey link…

-Done

Results:
Line 146: the term ‘primary care physician’ was earlier used, suggest to standardize throughout the manuscript.

-Done

Formatting of Table 2 can be improved.

-Done

Table 4: the first column described the ‘statements’. ‘Those who answered ‘agree’ and ‘strongly agree’ are the description for the second and third columns (‘N’ and ‘%’).

Done

Limitations of the study:
Line 260: The limitation of this study was the low response rate.

The low response rate and small sample size may restrict generalizability. Vaccination uptake was self-reported rather than verified with official records, introducing potential social desirability bias. Recall bias is also possible; however, the survey was conducted over three weeks during the flu season (October 2021).

Suggestion for the author to check the overall manuscript as there are some spelling mistakes to be corrected e.g. ‘health canter’ and to check the use of proper noun and common noun.

Done

Reviewer #2: Dear Author,

Thank you for addressing my concerns appropriately in this round of reviewing.
I think your study is very relevant in preventive health care
I wish you the best outcome.

7. PLOS authors have the option to publish the peer review history of their article ([what does this mean?](https://track.editorialmanager.com/CL0/https:%2F%2Fjournals.plos.org%2Fplosone%2Fs%2Feditorial-and-peer-review-process%23loc-peer-review-history/1/010f0199ff2bf550-1a75023e-f086-4322-8d7f-d2a752d9b63d-000000/UD8ditIyf_1pLR1NSdwVemd7R6EW_MNTqX9GpBr6IkQ=233)). If published, this will include your full peer review and any attached files.


If you choose “no”, your identity will remain anonymous but your review may still be made public.


**Do you want your identity to be public for this peer review?** For information about this choice, including consent withdrawal, please see our [Privacy Policy](https://track.editorialmanager.com/CL0/https:%2F%2Fwww.plos.org%2Fprivacy-policy/1/010f0199ff2bf550-1a75023e-f086-4322-8d7f-d2a752d9b63d-000000/sUiCQQJqFGoqoQ04CvdrVy1-Vc-zBxdNsvZux-LPqCw=233).

Reviewer #1: No

Reviewer #2: No
